# The unmet needs of gay and bisexual men following prostate cancer treatment: an integrative literature review

**DOI:** 10.1007/s11764-025-01934-x

**Published:** 2025-11-19

**Authors:** Obrey Alexis, William Garbrah, Aaron James Worsley

**Affiliations:** 1 Oxford Brookes University, Oxford, UK; 2 Open University, Milton Keynes, UK

**Keywords:** Prostate cancer, Gay, Bisexual, Unmet needs, Cancer care, Support groups, Healthcare professionals

## Abstract

**Purpose:**

Evidence has highlighted the unmet physical and psychological needs of men throughout all stages of prostate cancer (PCa); however, much of the focus has been on heterosexual men. Research shows that gay and bisexual men (GBM) have unique and unaddressed healthcare needs. The aim of this study is to systematically review the literature by exploring the needs of GBM following their treatment for prostate cancer.

**Methods:**

An integrative literature review sought qualitative and quantitative primary research from five databases (British Nursing Database, CINAHL, PsycINFO, PubMed, and Web of Science). A keyword search was employed, and each database was systematically searched. A thematic analysis was undertaken of relevant literature.

**Results:**

The evidence shows that GBM have unmet information, support, and sexual needs. GBM with PCa frequently reported a lack of tailored information about how treatment affected their sexual health. Many felt isolated as mainstream PCa support groups catered to heterosexual men. Some GBM felt unsupported by healthcare professionals (HCPs) who seemed to overlook their unique needs.

**Conclusions:**

GBM with PCa faces distinct challenges regarding sexual health, information access, social support, and interactions with HCPs. A more inclusive approach to healthcare practices, support services, and clinical care for GBM is required.

Implications for cancer survivors

HCPs should caution GBM about the potential sexual dysfunction post-treatment and avoid making heteronormative assumptions. Tailored information catered to GBM should be delivered, such as signposting to support groups specifically for GBM.

## Introduction

In 2022, approximately 1.5 million new cases of prostate cancer (PCa) were identified and about 400,000 deaths occurred worldwide [1]. At the time, PCa was recorded as the second most common cancer among men globally, with trachea, bronchus, and lung cancer being the most prevalent cancers in the world [1]. Gay and bisexual men (GBM) treated for PCa often experience disease and treatment-related side effects that negatively impact their quality of life, mental health, and sexual function [2]. However, psychological and social support lessen the impact for many GBM who are affected by post-PCa treatment [3]. Research has shown that efforts to improve outcomes for GBM with PCa have largely ignored their unique circumstances [4, 5].

GBM have historically experienced unique, unaddressed healthcare needs and obstacles [6]. Healthcare providers are seldom aware of issues affecting gay men differently from those of their heterosexual counterparts. In the healthcare sector, some oncology nurses believe that they will not be able to offer specific advice, support, and address the needs of GBM because they feel that they do not know enough about their lives, cultures, or sexual practices [6]. Arguably, some healthcare professionals (HCPs) lack the knowledge regarding issues impacting GBM and therefore signposting them to appropriate services such as peer support groups is warranted [4].

Most of the published literature regarding PCa has been conducted primarily with heterosexual, mostly married, Caucasian men [7]. The invisibility of the GBM in cancer care generally [8], and of GBM with PCa specifically, has been highlighted [7, 9]. GBM will experience the same treatment side effects as heterosexual men; however, the impact on GBM may be different and, in some ways, unique [2, 7, 10]. Studies based on GBM with PCa specifically highlight that they report worse quality of life, worse satisfaction with treatment, and worse psychological and cancer-related distress after treatment than their heterosexual counterparts [11]. Moreover, they experience increased discrimination [12] and significant cancer outcome disparities [2, 11].

A study conducted by Hart et al. [13] revealed that GBM experience significantly more urinary, bowel, physical and hormonal symptoms, and greater sexual dysfunction compared to heterosexual men. Moreover, the literature has also highlighted distressing psychosocial concerns facing GBM with PCa [14]. Relative to heterosexual men, GBM with cancer have reported greater depressive symptoms and relationship difficulties [15] including poor healthcare treatment [16].

Qualitative studies have described concerns such as heteronormativity in the healthcare system [17, 18]. For example, healthcare providers assume that patients engage in only heterosexual activities [17]. Moreover, GBM have described a lack of understanding by HCPs about the ways that they are differentially affected by sexual changes following treatment for PCa. Assessments of erectile function frequently presume vaginal and not anal penetration, which requires greater erectile rigidity; therefore, GBM have become frustrated by the lack of sensitivity to their needs [13] and reported lower satisfaction with PCa health care [16].

Studies exploring the needs of men throughout all stages of PCa have highlighted their unmet physical and psychosocial needs [19, 20]. GBM may need different social support and informational support, such as referrals to gay-friendly doctors. They may also require emotional support regarding erectile dysfunction or prostate sensitivity given the more prominent role that anal sex plays in gay men’s sex lives. Lower social support can contribute to worse PCa outcomes [21] and differences in social support may contribute to disparities in outcomes between GBM and heterosexual men [22, 23].

In the United Kingdom (UK), Stonewall [24] conducted the largest survey ever of GBM’s health needs and found that they were not being met. Significant areas of concern were identified noting that specific health needs for GBM were overlooked by HCPs. The survey provides evidence that GBM often felt neglected by a health system that has a duty to treat everyone equally [24, 25]. Furthermore, the European Cancer Organisation [26] reported discrimination, lack of culturally competent care, and limited access to services for GBM when accessing cancer care.

A systematic review [27] examined the existing research on patient-reported outcomes following cancer treatment among sexual and gender minority (SGM) populations. Similarly, a scoping review conducted by Kent et al. [28] explored the outcomes and experiences of SGM individuals diagnosed with cancer. While both studies provide valuable insights, their primary focus was on cancer in general rather than PCa specifically. Nonetheless, the findings from these reviews can offer important context for understanding the unique needs of GBM diagnosed with PCa. Building on this foundation, the aim of the present study is to systematically review the literature to explore the specific needs of GBM following treatment for PCa.

## Methods

This integrative review will synthesise the literature on the experiences of GBM following treatment for PCa. Qualitative, quantitative, and mixed methods primary research were included in this study. The review will adopt the framework described by Whittemore and Knafl [29] as a guide. This methodological approach allows for the simultaneous synthesis of qualitative and quantitative data.

### Search strategy

A scoping search found a low number of results across databases; therefore, literature was sought from the British Nursing Database, CINAHL, PsycINFO, PubMed, and Web of Science and without setting any date parameters so as to encompass a wide range of results.

The following keywords were used across all databases, searched in All Fields, with appropriate Thesaurus terms also included that were specific to that database, with the exception of Web of Science which does not contain Thesaurus terms:

(gay or homosexual OR bisexual OR “men who have sex with men” OR MSM).

AND

(prostat* cancer OR prostat* neoplasm* OR prostat* carcinoma).

AND

(post-treatment OR post treatment OR “following treatment” OR “after treatment”).

### Inclusion criteria

The inclusion criteria were studies published in any country, peer-reviewed research, and English language publications. Study participants included male, gay or bisexual, single or in a relationship, diagnosed with and treated for PCa, mixed methods papers, surveys, questionnaires, and quantitative papers.

### Exclusion criteria

The exclusion criteria were editorials, abstracts, opinion pieces, conference extracts, review papers, dissertations, secondary analyses, meta-syntheses, literature reviews, non-English language papers, studies incorporating other types of cancer, and studies that included heterosexual men and transgender women.

**Fig. 1:**
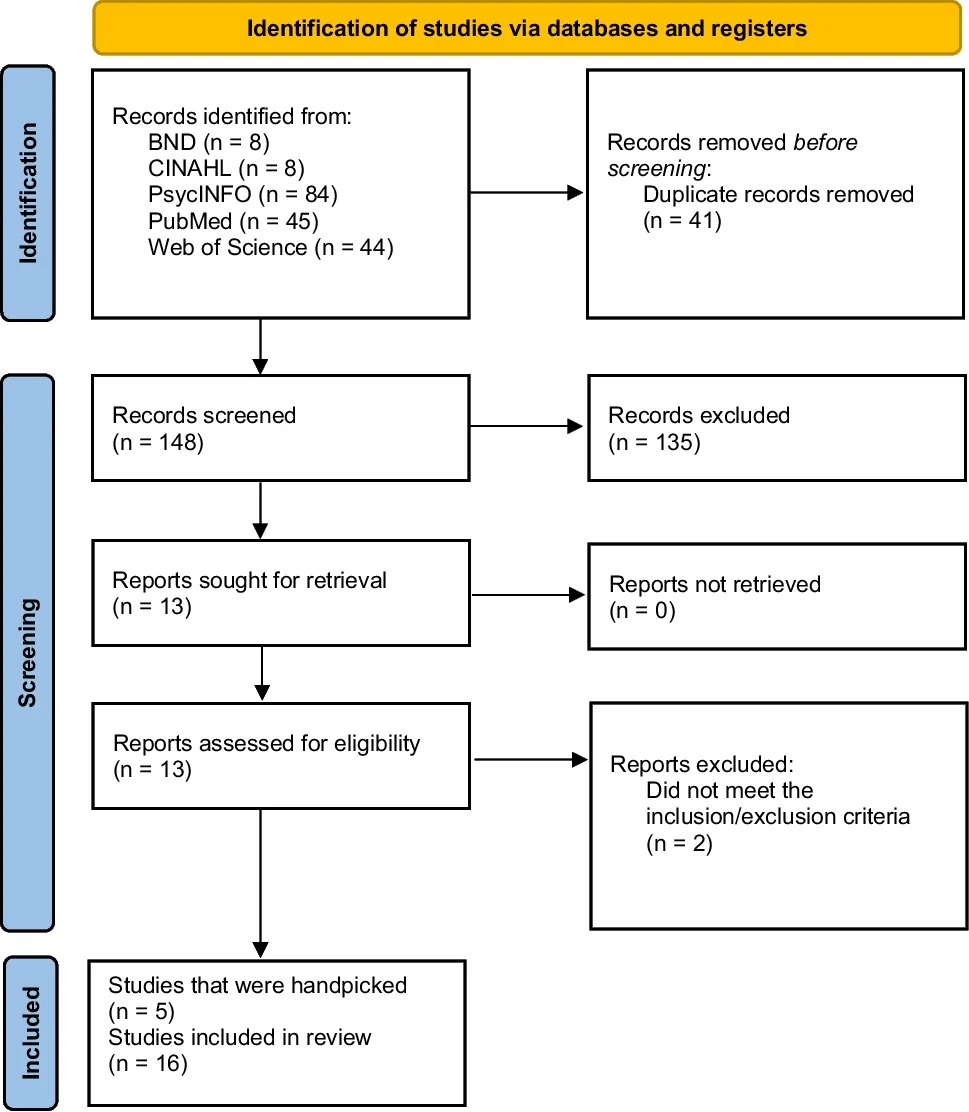
PRISMA flow diagram[30]

The search results can be seen in Fig. 1. The results were uploaded into Rayyan and then screened by all three authors by title and abstract. Any disputes were deferred to a third author to make a final decision. Out of 148 papers, 13 were initially deemed eligible for this review. A further 5 papers were found through hand searching references of relevant papers. These chosen papers were then read in full length by the authors, and 2 were excluded for not meeting the inclusion criteria. In total, 16 papers were selected for data extraction.

### Quality appraisal

The Mixed methods appraisal tool (MMAT) which is a critical appraisal tool designed for the appraisal stage of systematic mixed studies reviews [31] was applied to the chosen literature to assess its quality. The studies for this integrative review all scored marks that ensured their inclusion. Out of the 16 studies selected for this integrative review, 7 were quantitative, 6 used a qualitative approach, and 3 were mixed methods.

The authors reread these 16 papers, and data were extracted from the papers and added to individual cells in Google Sheets. This enabled the authors to find common meanings, similarities, and differences amongst the data, as described by Pfaff et al. [32]. Subsequently, the authors were able to discern a number of themes emerging from the codes. The authors then discussed and agreed on these codes, before grouping them into major themes.

## Results

A total of 16 studies were eligible for this review (Table 1). The studies were published from 2013 to 2024 and were conducted across the globe: United States of America (USA) (*n* = 7), Australia (*n* = 2), UK (*n* = 2), Canada (*n* = 2), Sweden (*n* = 1), and two with international samples (USA, Canada, UK, and New Zealand). Most were qualitative research studies (*n* = 7) and the remaining employed quantitative research (*n* = 6) or mixed methods (*n* = 3). The number of participants ranged from 8 to 558 (see Table 2).

The needs of GBM following treatment for PCa are described in four main themes: (i) sexual needs; (ii) information and resource needs; (iii) social and emotional support; and (iv) support from HCPs.

### Sexual needs

GBM reported experiencing a reduction in or cessation of sex, with loss of sexual activity described as devastating [17, 33–35]. Participants indicated that, following PCa treatment, the existing tools for measuring sexual function in gay men primarily focus on erectile function. However, for gay men who engage in receptive anal intercourse, sexual pleasure may be derived from non-genital means and therefore the current sexual function questionnaires may not fully capture their experiences or may assess sexual function only partially [36]. Gay men were concerned about how each treatment might affect aspects of their sexual activity, which, in turn, could also affect their partners [12, 35, 37, 38]. They were particularly concerned about a reduction in penis size, erection problems, and urinary incontinence as they found it both embarrassing and grievous [34–36]. For men who were sexually active within the gay community, experiencing erection problems was distressing [34, 35, 39], given the perceived emphasis on youth, masculinity, body image, and sex in the gay community [34, 35]. Some men were concerned that their PCa would render them less valuable within the gay community [17, 34, 35] and were unable to compete with purported healthier men [17, 34]. Similarly, other studies [34, 35, 37, 39] argued that most GBM rated their quality of life and sexual functioning as highly problematic, indicating that sexual recovery of GBM after PCa is possible, but very challenging [37–39]. It was further reported that the sexual effects of PCa treatment are much broader than only erectile dysfunction [34, 39]. For example, men who had identified as gay in later life reported a double jeopardy: the loss of sexual functioning and loss of further opportunity to explore recently discovered sex with other men [34]. Other concerns included the effects on receptive anal sex, urination in sex, and change in role in sex, such as from receptive to insertive [34, 38, 39]. Clinicians were encouraged to discuss all these concerns with GBM following PCa treatment [37–39]. Patients may be overly optimistic about their chances of sexual recovery even when fully forewarned. It is therefore equally important for HCPs to caution GBM about the potential sexual dysfunction post-treatment [37, 38].

The risk of developing sexually transmitted infections (STI) such as HIV was a major concern for GBM following PCa treatment, particularly for those GBM who received surgery. Very few GBM who engaged in insertive anal sex reported using condoms, with most citing erection difficulties as the underlying reason for non-condom use [37]. As GBM are prone to developing STIs following PCa treatment as a result of non-condom use, it is imperative for HCPs to discuss alternatives to condoms, such as pre-exposure prophylaxis (PrEP) and referral to a PrEP provider for HIV-negative patients, and review of antiretroviral medication adherence for HIV-positive ones [37]. Additionally, HCPs must highlight the risk of erectile difficulties leading to HIV/STI transmission either through change in role in sex or non-condom use needs to be raised with patients [37].

GBM received an earlier diagnosis of PCa than heterosexual men due to knowing the importance of getting tested; however, the loss of ejaculation was reported to be more common among GBM following treatment for PCa than in heterosexual men [39]. Moreover, the loss of ejaculation was more distressing for GBM than heterosexual men [4, 35, 36, 39]. Due to erectile dysfunction, the number of males who practised insertive intercourse declined following PCa treatment [7, 35]. Many non-heterosexual men continued to be sexually active by switching from insertive to receptive anal intercourse [7, 35].

### Information and resource needs

Gay men with PCa have unmet information needs [33, 34] and discussed the need for HCPs to provide them with information about different treatment options and their potential risks, allowing these patients to weigh the risks and benefits in making a clinical decision [33, 35, 36, 40, 41]. While there are many information pamphlets and support groups for PCa patients, these are not necessarily targeted to or inclusively for GBM [40]. Allensworth-Davies et al. [9] and McConkey and Holborn [35] argue that providing opportunities for GBM to speak openly and in a safe environment about their treatment concerns and changes in sexual function could boost post-treatment quality of life for GBM.

The participants wanted information about the implications of PCa that was specifically designed to meet the needs of GBM, but they found that this was not available [17, 40, 41]. The unavailability of such information directly relevant to GBM in the educational material provided within clinics and online made the participants feel that they were poorly informed and left out [17]. Specifically, gay men noted that the little information available about resuming sex after treatment, was ambiguous on whether this only applied to those who engaged in vaginal intercourse [17, 40]. Gay men also expressed concerns pertaining to sources of information concerning medication and devices to aid or enhance their sexual function. They were reluctant to discuss the situation with their healthcare providers and therefore relied on internet sources [17, 35, 40]. Although they were nervous about the efficacy of such medication, they considered this his only option as they felt that their unique needs were not acknowledged [17].

GBM felt that some of the diagnostic and assessment instruments had not been validated for use among these patient groups [4].For example, Lee et al. [42] noted the need for validated questionnaires targeted to the GBM population as current instruments presumed all clients to be heterosexual. They therefore expressed the need for a validated instrument tailored for and directed to measure sexual satisfaction among GBM [4, 36, 42]. Such instruments could facilitate clinicians to better engage with GBM who practice receptive anal intercourse [36, 42]. Conversely, the lack of these instruments made GBM feel uncared for after treatment and did not help chart the trajectory of their sexual recovery pertaining to receptive anal intercourse following PCa treatment [36].

### Social and emotional support

The need for peer support and group support, to help navigate the physical and emotional challenges associated with treatment-related sexual dysfunction, was a major source of concern for GBM [35, 41]. To find first hand experiences and to aid in their clinical decision-making process regarding PCa treatment, some GBM sought out social support groups to find information about possible treatments and side effects of PCa [4, 35]. Some of the studies reported that most of the GBM felt the need for social and emotional support specifically designed for GBM following treatment of PCa [4, 35, 36, 40]. Although several men engaged with both online and in-person support groups during this phase of their PCa experience, some men felt disappointed as a result of the lack of access to GBM support groups locally [4, 7, 35]. They felt that having such in-person support groups could help them be more at ease when talking with other GBM who understand their needs [4, 7, 35, 36]. In contrast, participants who discovered GBM support groups in their local areas described the relief they felt from connecting with people who were in similar situations. Most of these groups were identified through their personal network or online as opposed to signposting within the healthcare services. Although participants had to travel considerable distances to attend such groups, they felt the support and information they gained from these groups was worth the journey [17, 35].

Several men disclosed that their PCa diagnosis triggered emotional and psychological distress that required support from HCPs and loved ones [17, 35, 39]. Some were concerned about their mortality and the association of the disease with their past, and specifically their sexuality or coming‐out experience [17, 35]. Others also felt stigmatised due to their sexual orientation [35, 38, 39] and magnified their risk associated with poor mental health [37, 39]. At the same time, most of these men reported feeling lonely during medical appointments as most of them visited the clinics alone [17]. This lack of social support for GBM was exacerbated by their sexual orientation as those who had partners were uncertain about whether same‐sex partners would be welcomed by healthcare staff [17, 35, 40]. Lee et al. [40] therefore suggested the need to incorporate GBM sexual practices and preferences into clinical decision making, particularly oncological and psychosocial support services that must be tailored to accommodate the needs of GBM. This was also echoed by Mehta et al. [41] highlighting the need for HCPs to emotionally prepare the patients.

GBM experience a decline in health-related quality of life after PCa treatment which resulted in increased rates of psychological and emotional distress [35, 36]. Whereas some participants obtained emotional support from family members [4, 35], others expressed the need to have social support [4, 40]. For example, having people attend appointments with them [4]. The men also reported being selective about their sexual life and the sexual side effects of their treatment. Some confided in partners, others in friends or other social support groups but generally less in family [4, 35]. The participants also expressed the need to have loved ones more involved in the sexual recovery process and they appreciated when their physicians had involved their partners in the discussion about PCa treatment and treatment-related expectations [41]. Additionally, Mehta et al. [41] further observed that both patients and partners expressed an interest in improved communication with each other. They were willing to share their concerns about treatment-related sexual side effects, and to understand each other’s needs regarding the physical loss of sexual function. Furthermore, partners were eager to discuss emotional changes associated with the onset of sexual dysfunction [35, 41].

### Support from HCPs

The need for clinicians to refer GBM to local groups and/or to online groups was highlighted by several participants [4, 17, 40]. The importance of thorough counselling by HCPs regarding the sexual side effects of PCa treatment was highlighted by GBM [7, 17, 36, 41]. They expected honest discussions with HCPs, about how their condition could impact their activities of daily living, including their sexual activity [7, 9, 17, 40, 41]. On the other hand, several studies [33, 41, 43] reported that physicians may be unwilling to discuss the sexual side effects of treatment for fear that patients would not accept the treatment due to the potential side effects. It must be acknowledged that generally gay men felt more comfortable disclosing their sexual orientation to nurses (mostly women) compared to doctors who were mainly men [17, 40]. On the contrary, McConkey and Holborn [35] argued that GBM reported positive experiences with their General Practitioners (GP) to whom they were all out, as they served as an important source of support. Also, gay men, particularly those who were older [17], reported being reluctant or embarrassed to disclose their sexual orientation to their HCPs [17, 40, 42–44]. The sense of difference and isolation was compounded when it came to discussing sex and partnerships [33–35, 40, 42, 43]. On the other hand, younger HCPs compared to older HCPs were generally perceived to be more receptive about gay issues [17, 33]. It is worrying for gay men to notice that not only did HCPs appear to be disinterested in issues related to gay men [17, 34, 42, 44], but they also lacked empathy about the participants’ situation or engaged with them during consultations [17, 43]. Some respondents felt that HCPs were homophobic or prejudiced about their sexuality and, on some occasions, offensive [17, 43].

Another major concern raised by the participants was the perceived lack of knowledge on the part of the healthcare team about the needs of GBM [17, 34, 40, 44] and how their experience differed due to their sexual orientation [17, 33, 40, 41, 43]. GBM described their communication with HCPs as cancer-focused and medically driven, as it was perceived as negating sexual well-being and the impact of PCa and its treatment on sexuality and sexual practices [33, 34, 38, 43]. The current knowledge gap is attributed to the lack of historical acceptance, prejudice in the medical community, and psychosocial barriers in the gay community [42, 43]. GBM were particularly disappointed, as the information provided by the clinicians was perceived as being misplaced or influenced by heteronormative assumptions [17, 34, 43]. Some studies [7, 17, 40–43] suggested that HCPs should be given training and support to enable them to provide information and support that is culturally relevant. They further suggested that HCPs should consider requesting the sexual orientation of patients when gathering information [17, 37, 42, 44]. This is particularly important as PCa remains one of the most commonly diagnosed malignancies among men and therefore potentially includes a large population of GBM; thus, the disclosure of sexual practices could help increase the recruitment of GBM into related research [42, 43].

## Discussion

The evidence shows that sexual recovery is a major problem for GBM who have both been treated for PCa and have had their prostate removed. From the literature, it is clear that PCa and its treatments such as surgery, radiation, and hormone therapy can significantly impact sexual function, leading to erectile dysfunction, loss of libido, and changes in orgasm [37]. For GBM, these changes may uniquely affect their sexual identity and relationships, especially if they engage in receptive anal intercourse, which can be compromised due to treatment side effects [2]. The loss of ejaculation and changes in penile sensitivity can also alter intimate experiences rendering GBM to feel inadequate [36].

The National Institute for Health and Care Excellence (NICE) [45] stipulates that men with PCa are provided with person-centred information tailored to their needs by a senior medical doctor or specialist nurse. Moreover, NICE [45] recommends that information and support resources should be checked to ensure that the information is clear, reliable and current. From this review, GBM with PCa frequently report a lack of tailored information about the specific ways treatment affects their sexual health and relationships [17]. Additionally, this review found that GBM treated for PCa wanted HCPs to offer information regarding treatment options and its potential risks so that they can assess the risks and benefits to them [41]. The review also reveals that most educational materials adopted a heterosexual perspective, leaving out discussions on how PCa affects anal sex, partner dynamics in same-sex relationships, and the psychological impact on GBM’s identity [35]. GBM were reported to use the internet to seek more tailored information [17, 35, 40]. Organisations such as Prostate Cancer UK [46] do offer online advice on PCa for GBM, but there is a risk GBM may encounter misinformation from other sources.

GBM with PCa often experiences unique social challenges, such as fear of rejection from partners or a loss of connection to GBM communities due to altered sexual function and loss of support from support groups [41]. In the UK, there are many support groups for men with PCa. Most of these groups were unable to meet the needs of GBM. The focus of these groups was on heterosexual men and not on GBM and therefore GBM found it challenging to raise issues associated with their sexual identity for fear of causing embarrassment to others [17]. Similarly, many feel isolated because mainstream PCa support groups cater to heterosexual men and may not be welcoming or inclusive of discussions around same-sex relationships and sex [35]. There are independent support groups in the UK for GBM, such as LGBT Walnut and Macmillan LGBT lounge, which offer specific support and information for GBM, but these are either based in major cities, such as London and Manchester, or are hosted online. Other examples are MaleCare in the USA, Shine a Light in Australia, and GBTQ Prostate Cancer Support Group in Canada. The evidence shows that peer support networks tailored to GBM cancer survivors can provide emotional reassurance and guidance in navigating post-treatment life [4].

GBM have postulated that the current medical and healthcare approach is strongly focused on heteronormative patients [18, 47]. Due to the lower numbers of GBM who are treated for PCa, HCPs often assume that patients are heterosexual without confirming the sexual orientation of their patients. Further, some men reported experiencing heteronormative assumptions. For example, even if the HCPs are aware of the patient’s sexual orientation, their care, support, treatment and needs are planned in the same way as heterosexual patients [33]. Healthcare providers may lack cultural competence in addressing the specific needs of GBM with PCa [47]. A review by Kent et al. [28] underscored the need to advance equitable healthcare for SGM populations. In a similar vein, a systematic review [27] emphasized the importance of clinician education to support individualized care grounded in patient values and priorities. This includes integrating considerations of sexual orientation, gender identity, and sexual practices, rather than relying on reductive, binary gender markers. Such an approach fosters inclusive, person-centred medical decision-making. Recognizing the complexity of SGM identities is essential to clinical competence. Ultimately, equity in care requires HCPs to be responsive to diverse lived experiences.

GBM felt unsupported by HCPs, particularly doctors, and perceived that they were embarrassed to discuss issues associated with sexual orientation and sexual function [17]. HCPs need to be aware of the broad implications of sexual dysfunction on GBM treated for PCa and its impact on their quality of life which was perceived to be treated with the same importance and concern as their heterosexual counterparts [40]. Similar findings were found in studies conducted by Motofei et al. [10] and Hart et al. [13] where information and the sexual needs of GBM were overlooked by HCPs thus leaving them frustrated with their interaction with HCPs and somewhat emasculated.

For many men, sexual function is closely tied to their sense of masculinity. Previous evidence has shown that gay men are affected by notions of stereotypical masculine ideals [48]. Erectile dysfunction, loss of libido, and changes in orgasm can lead to feelings of emasculation and insecurity [13]. In heterosexual contexts, masculinity is often reinforced through penetrative sex, but for GBM, the loss of the ability to engage in anal sex either as the receptive or insertive partner can profoundly affect self-image and intimate relationships [2]. Some men fear being seen as less desirable or less of a man within the gay community that emphasizes sexual performance and physical appearance [47]. Moreover, PCa may challenge traditional views of masculinity, and some men may find opportunities to redefine what masculinity means to them. For others this may involve shifting their focus from physical performance to emotional intimacy in relationships. Others may engage in peer support groups or therapy to develop a broader, more holistic understanding of masculinity—one that embraces vulnerability, self-care, and resilience [9, 35].

## Implications for cancer survivors

The current review indicates that sexual function in GBM particularly as it relates to receptive anal intercourse is measured wrongly following PCa treatment. These findings are consistent with a prior study showing greater levels of sexual activity in gay men compared with heterosexual men [49]. The current review strengthens an observation made by previous researchers [2, 16] that GBM experience a decline in health-related quality of life post PCa treatment. There is evidence of psychological stress resulting from erectile dysfunction which may be attributed to a decline in sexual activity after PCa treatment [40, 50]. Patients may be overly optimistic about the possibilities of remaining sexually active post their treatment, even when fully forewarned. HCPs should therefore caution GBM about the potential sexual dysfunction post-treatment. As reviewed by this study, it is also well established in previous literature [40, 50] that perceived emphasis on youth, masculinity, body image, and sex in the gay community often plays an important role in the identities of gay men.

HCPs should avoid heteronormative narratives when caring for GBM with PCa. Training and resources within healthcare services can help enhance cultural competencies and equity. Knowledge of support groups for GBM, or initiating GBM support groups, is integral to allow GBM a safe space for discussion. Policies and guidelines should focus on the salient psychological and psychosexual health of GBM PCa patients, emphasizing the unique needs of GBM. Urologists must be prepared to have frank and open discussions with GBM patients. The international Guidelines for Sexual Health Care for Prostate Cancer Patients [51] provide clinicians with a comprehensive approach to sexual health care for GBM patients with PCa.

Research into PCa has traditionally focused on heterosexual men, leaving significant gaps in understanding the unique experiences and needs of GBM globally. As societal acceptance and visibility of LGBTQ + individuals increase worldwide, it becomes crucial to address the disparities in PCa care and outcomes for GBM [47]. Globally, GBM with PCa face distinct challenges, including stigma, limited culturally competent healthcare, and a lack of tailored information. Sexual functioning after treatment is a primary concern, yet existing research often overlooks how sexual side effects—such as erectile dysfunction or changes in ejaculation—affect men who have sex with men differently [37, 52]. For example, the impact of PCa on anal intercourse or the dynamics of same-sex relationships remains underexplored. This indicates a critical need for studies that include diverse sexual practices and identities in their design.

Healthcare systems in many countries do not routinely collect data on sexual orientation, creating a barrier to understanding prevalence, outcomes, and psychosocial impacts specific to GBM [34]. Research must therefore advocate for inclusive data collection policies and practices globally, enabling more accurate assessments and interventions. Furthermore, in countries where homosexuality is criminalized or highly stigmatized, GBM may be less likely to seek medical help, let alone participate in research. This underscores the need for ethically sensitive and culturally appropriate methodologies when conducting international studies.

Another implication is the need to examine disparities in survivorship support [4]. GBM may lack traditional support systems—like a spouse or children—and may instead rely on chosen families. Research should investigate how these networks influence coping mechanisms and access to care. Additionally, mental health outcomes for GBM with PCa are under-researched, particularly given the intersection of sexual minority stress and cancer-related distress.

Research must consider the development of tailored sexual rehabilitation programs, partner-inclusive care models, and educational resources that reflect the diversity of GBM experiences. Multinational collaborations and research can help overcome national and international challenges and promote equity. Therefore, addressing the specific needs of GBM with PCa from a global perspective requires inclusive research design, culturally competent methodologies and a commitment to increasing the critical mass in an area that is under researched.

Moreover, balancing the need for data collection on PCa among GBM with the rise of anti-LGBTQI policies in the USA requires ethically sensitive, community-centred methodologies that prioritize participant safety, privacy, and trust. Researchers must design studies that minimize risk by using secure, anonymous data systems, carefully worded consent processes, and recruitment strategies that avoid outing participants or linking data to identifiable information. Collaborating with LGBTQI health organizations and advisory boards helps ensure cultural competence, relevance, and ethical oversight. Methodologies should emphasize trauma-informed, affirming approaches that acknowledge systemic discrimination while ensuring that findings serve to reduce health disparities rather than reinforce stigma.

## Conclusion

This review contributes to the growing body of evidence on PCa in GBM by highlighting the unique sexual, psychological, and social challenges faced by this group following treatment. It demonstrates how sexual dysfunction—such as erectile difficulties, loss of ejaculation, and changes in orgasm—can significantly disrupt GBM’s sense of identity, particularly in relation to same-sex intimacy and roles in anal sex. The review exposes the pervasive heteronormativity in healthcare delivery, noting that information, treatment planning, and support services are often designed for heterosexual men, leaving GBM underserved and marginalised. It underscores the lack of culturally competent care and tailored educational materials, leading many GBM to seek potentially unreliable information online. The inadequacy of mainstream PCa support groups is also explored, alongside the value of GBM-specific peer support networks. Additionally, the review offers insight into how gay and bisexual men experience disruptions in masculinity post-treatment and may seek to redefine identity through emotional intimacy, resilience, and community support. These findings underscore the urgent need for inclusive, affirming, and person-centred care for GBM living with and beyond PCa.

Furthermore, GBM with PCa face distinct challenges regarding sexual health, information access, social support, and interactions with HCPs. Addressing these disparities requires a more inclusive approach to healthcare practices, support services, and clinical care to ensure all men receive the guidance and support they need during and after PCa treatment. Moreover, HCPs should encourage open conversations and allow GBM to express their changing perceptions of self-worth and intimacy. Additionally, person-centred care is the goal that HCPs aim to achieve and therefore allowing GBM to express their needs in a non-threatening environment will ultimately enhance their care and treatment.

**Table 1:** Mixed methods appraisal of the studies

1. Qualitative	Capistrant et al., (2016)	Doran et al., (2018)	Jagervall et al., (2019)	Lee et al., (2015)	McConkey and Holborn., (2018)	West et al., (2021)
1.1. Is the qualitative approach appropriate to answer the research question?	Y	Y	Y	Y	Y	Y
1.2. Are the qualitative data collection methods adequate to address the research question?	Y	Y	Y	Y	Y	Y
1.3. Are the findings adequately derived from the data?	Y	Y	Y	Y	Y	Y
1.4. Is the interpretation of results sufficiently substantiated by data?	Y	Y	Y	Y	Y	Y
1.5. Is there coherence between qualitative data sources, collection, analysis and interpretation?	Y	Y	Y	Y	Y	Y

4. Quantitative descriptive	Allensworth-Davies et al., (2013)	Amarasekera et al., (2020)	Dowsett et al., (2014)	Lee et al., (2013)	Simon Rosser et al., (2019)	Wassersug et al., (2013)	Wheldon et al., (2022)
4.1. Is the sampling strategy relevant to address the research question?	Y	Y	Y	Y	Y	Y	Y
4.2. Is the sample representative of the target population?	Y	Y	Y	Y	Y	Y	Y
4.3. Are the measurements appropriate?	Y	Y	Y	Y	Y	Y	Y
4.4. Is the risk of nonresponse bias low?	Y	Can’t tell	Can’t tell	Can’t tell	Can’t tell	Can’t tell	Can’t tell
4.5. Is the statistical analysis appropriate to answer the research question?	Y	Y	Y	Not enough data collected	Y	Y	Y

5. Mixed Methods	Mehta et al., (2019)	Rose et al., (2017)	Ussher et al., (2017)
5.1. Is there an adequate rationale for using a mixed methods design to address the research question?	Y	Can’t tell	Y
5.2. Are the different components of the study effectively integrated to answer the research question?	Y	Y	Y
5.3. Are the outputs of the integration of qualitative and quantitative components adequately interpreted?	Y	Y	Y
5.4. Are divergences and inconsistencies between quantitative and qualitative results adequately addressed?	Y	Y	Y
5.5. Do the different components of the study adhere to the quality criteria of each tradition of the methods involved?	Y	Y	Y

**Table 2:** Extracted data from original studies with quality rating scores

Author(s) (year), country	Aim/purpose of study	Methods/instruments/participants	Key findings
Allensworth-Davies et al. (2016) (USA)	To identify factors associated with masculine self-esteem in gay men following treatment for localized PCa and to determine the association between masculine self-esteem, PCa-specific factors, and mental health factors in these patients	A quantitative cross-sectional survey of gay PCa survivors (*n* = 111) with questionnaire	Stigma experienced by GBM as part of their sexual orientation, magnified the risk associated with poor mental health among gay elders. This finding suggests that open communication with one’s PCa physician may be an important factor in a gay man’s recovery from PCa. Given that few PCa support groups exist specifically for gay men, increased opportunities for speaking openly and in a safe environment about treatment concerns and changes in sexual function could benefit post-treatment QoL for GBM
Amarasekera et al. (2020) (USA)	To determine if there are sexual function domains relevant to gay men that are not captured by the Expanded Prostate Cancer Index Composite (EPIC) sexual function assessment	Quantitative online survey regarding the applicability of the sexual function domain in the validated EPIC question with Gay men (*n* = 53) diagnosed with PCa	Sexual function in gay men relating to receptive anal intercourse is measured incompletely when assessing sexual function after PCa treatment. There is a decline in health-related quality of life after PCa treatment in GBM and increased rates of psychological distress from erectile dysfunction
Capistrant et al. (2016) (USA)	To investigate the availability and provision of social support for gay and bisexual men with prostate cancer (GBMPCa)	A qualitative data from in‐depth, semi structured, one‐on‐one telephone interviews of GBM (*n* = 30) with PCa. Inductive and deductive codes were used to identify themes about social support provided to GBMPCa during diagnosis and treatment	Participants expressed wanting more in‐person support groups specifically for GBMPCa to make them feel more at ease when talking with other gay men. The participants also expressed the need of having people attend appointments with them. They wanted to hear directly from first hand experiences and to aid in their own decision-making process regarding treatment. Men were selective as to whom they talked to regarding sexual side effects
Doran et al. (2018) (UK)	To highlight the needs of gay men with prostate cancer, and their experiences of healthcare provision	A phenomenological research method was used to collect and analyse data. In‐depth interviews were conducted with gay men (*n* = 12) who had been diagnosed with prostate cancer	Participants wanted, and expected, candid discussions with HCPs, about how prostate cancer could affect their lives, sexual function, and how to access culturally relevant support before and after treatment. Information provided to GBM was perceived as being misplaced or informed by heteronormative assumptions. Information concerning medication and devices to aid or enhance sexual function was frequently sought using the internet as participants were reluctant to discuss their concerns with their healthcare provider. Participants described the need for male health professionals to show sensitivity and understanding by offering information and advice
Dowsett et al. (2014) (Australia)	To explore forms of sexual practice engaged in by men following treatment for PCa	An international online survey was conducted in 2010–2011 of men treated for PCa, where participants (*n* = 558) were asked explicitly about their sexual practices before and after PCa treatment	The number of males practicing being the insertive partner declined with PCa treatment due to erectile dysfunction. Many nonheterosexual men continued to be sexually active by switching from insertive to receptive
Jägervall et al. (2019) (Sweden)	To determine specific concerns of gay men’s post-prostate cancer treatment sexual practices	A qualitative study design was deployed using semi-structured interviews. self-identifying gay men (*n* = 11) aged 58–81 years and treated for PCa were interviewed	The need to emphasise the value of distinguishing between sexual identity and sexual practice when designing rehabilitation programmes for GBM with PCa should be considered by healthcare professionals. Men diagnosed with PCa identifying as gay or bisexual experience lacking sexual communication in interactions with health professionals
Lee et al. (2015) (Canada)	To explore post-PCa treatment sexual concerns for a sample of men-who-have-sex-with-men (MSM)	Qualitative methodology with individual semi-structured interviews of MSM (*n* = 16) either face-to-face or via Internet-based video conferencing. Participants were asked open-ended questions about their experiences of sexual QoL following PCa	The need to incorporate GBM sexual practices and preferences specifically into treatment decisions. The need for psychosocial support, information pamphlets and support groups targeted to or inclusively for MSM. Some men were also uncomfortable disclosing their sexual orientation in PCa support group settings
Lee et al. (2013) (Canada)	To evaluate post-treatment QoL in PCa patients who are MSM, and to investigate the utility of current QoL assessment tool	Qualitative study involving PCa patients treated with surgery and/or radiation were recruited from the local MSM community. Each participant completed the Expanded Prostate Cancer Index Composite (EPIC) questionnaire, Male Sexual Health Questionnaire (MSHQ), and a questionnaire focused on insertive and receptive roles of anal intercourse	The need for validated questionnaires targeted to GBM population as current instruments assessing sexual QoL may not be applicable or valid for PCa patients who have sex with men. Some medical professionals may continue to feel uncomfortable treating MSM. The lack of historical acceptance, continued perceptions in the medical community, and psychosocial barriers in the MSM community are challenges that have contributed to the current knowledge gap
Mehta et al. (2019) (USA)	To investigate what prostate cancer survivors and their partners want from a sexual recovery intervention, and whether they consider an online tool acceptable for use in promoting sexual recovery	A mixed-methods study with focus groups and interviews. Participants included both heterosexual and gay cancer survivors, as well as their partners	Lack of knowledge about the sexual side effects of PCa treatments, due to their healthcare provider focusing on cancer control only, rather than the impact of cancer therapies on long-term quality of life. physicians may be unwilling to discuss the sexual side effects of treatment for fear that patients would be unwilling to undergo cancer treatment due to the potential side effects. The need for better pretreatment counseling and emotional preparation about side effects and realistic expectations for recovery
McConkeya and Holbor, (2018) (UK)	To explore the lived experience of gay men with PCa	A qualitative methodological approach employing phenomenological method was used to collect and analyse data from eight (8) gay men treated for PCa	Gay men with PCa have unmet information and supportive care needs. The need for information related to the impact of sexual dysfunction and associated rehabilitation, negatively impacting their quality of life. Some men were uncomfortable with their healthcare providers due to issues associated with heteronormativity, minority stress, and stigma. There is the need for healthcare providers to update PCa education programmes to cater for the needs of GBM
Rose et al., (2016) (USA, Canada, UK, and New Zealand)	To examine GBM’s experiences of sexual communication with HCPs since the onset of PCa	A mixed method study comprising of a survey of GBM (*n* = 124) with PCa and male partners (*n* = 21), and interviewed a sub-sample of GBM (*n* = 46) and male partners (*n* = 7)	GBM perceived their communication with HCPs as challenging. Medical support was prioritised over sexual and psychological support. HCPs assumed; heterosexuality of GB patients making it difficult for the patients to disclose their sexual orientation. GBM perceived rejection or lack of interest and knowledge from a majority of HCPs with regard to gay sexuality and the impact of PCa on GBM
Rosser et al. (2019) (USA)	To assess the effects of treatment of PCa on GBM’s sexual behaviour	An online survey of gay and bisexual men with prostate cancer (*n* = 193) were recruited from North America's largest online cancer support group. Sexual functioning was measured using the Expanded Prostate Cancer Index Composite (EPIC) and a tailored Gay Sexual Functioning Inventory (GSFI)	GBM participants scored significantly worse on overall mental health compared to published results for other PCa survivors. The risk of HIV and STI after PCa treatment was a major concern for those GBM who received surgery as few GBM who engaged in insertive anal sex reported using condoms, with most citing erection difficulties as the underlying reason for non-condom use. Clinicians asking about sexual orientation could improve clinical practice
Ussher et al. (2016) (Australia)	To examine the meaning and consequences of erectile dysfunction and other sexual changes in GBM with PCa and male partners	A combination of on-line survey and one-to-one interviews was used to examine the meaning and consequences of sexual changes after PCa for GBM (*n* = 124) and male partners (*n* = 21)	Erectial dysfunction associated with reports of emotional distress, negative impact on gay identities, and feelings of sexual disqualification was reported by the participants. Feelings of exclusion from a sexual community central to GBM’s lives due to sexual challenges associated with their treatment of PCa
Wassersug et al. (2013) (USA, Australia, Canada, UK, and New Zealand)	To determine if heterosexual and nonheterosexual men treated for PCa differ in diagnostic and treatment outcomes and in various measures of physical health, sexual function, and wellbeing, before and after the treatment	Self-identified heterosexual (*n* = 460) and self-identified nonheterosexual men (*n* = 96) completed an online survey. The men in the 2 groups were then compared using logistic regressions that controlled for differences among countries	Non-heterosexual men received a diagnosis of PCa at an earlier cancer stage than heterosexual men, due to knowing the importance of getting tested. There was no difference in PC treatments or side effects of urinary incontinence or erectile dysfunction for non-heterosexual and heterosexual men. The only area where both populations diverged was in the loss of ejaculation, with non-heterosexual men finding this more distressing
West et al. (2021) (USA)	To investigate the experience of communicating sexual orientation by GBM and MSM to physicians involved in their prostate cancer care	A qualitative analysis conducted on GBM (*n* = 30) with in-depth interviews	Participants expressed difficulty in disclosing being GBM with their doctors as. GBM with PCa feel that their relationship with their specialist(s) is extremely important and need to be able to trust their physicians
Wheldon et al. (2022) (USA)	To explore the impact of prostate cancer and its treatments on RAI among GBM	A quantitative cross-sectional online survey of GBM prostate cancer survivors (*n* = 100) who reported pleasurable RAI prior to treatment	23% of the men who had attempted recent RAI reported having Anodyspareunia which was negatively associated with mental health, performing oral sex on a partner, and bowel function. The overwhelming majority received no information from their healthcare providers about loss of RAI function prior to prostate cancer treatment. Culturally responsive cancer survivorship care may need to address the loss of RAI function for GBM PCa survivors

## Data Availability

No datasets were generated or analysed during the current study.
